# Little Brain, Big Expectations

**DOI:** 10.3390/brainsci10120944

**Published:** 2020-12-07

**Authors:** Rubens Gisbert Cury, Carina França, Egberto Reis Barbosa, Manoel Jacobsen Teixeira, Daniel Ciampi de Andrade

**Affiliations:** 1 Movement Disorders Center, Department of Neurology, School of Medicine, University of São Paulo, 01000-000 São Paulo, Brazil; rubens_cury@usp.br (R.G.C.); egbertob@8415.com.br (E.R.B.); 2 Functional Neurosurgery Division, Department of Neurology, School of Medicine, University of São Paulo, 01000-000 São Paulo, Brazil; manoeljacobsen@gmail.com (M.J.T.); ciampi@usp.br (D.C.d.A.); 3 Service of Interdisciplinary Neuromodulation (SIN), Laboratory of Neurosciences (LIM-27), Department and Institute of Psychiatry, University of São Paulo, 01000-000 São Paulo, Brazil

**Keywords:** ataxia, cerebellum, dystonia, neuromodulation, Parkinson’s disease

## Abstract

The cerebellum has been implicated in the mechanisms of several movement disorders. With the recent reports of successful modulation of its functioning, this highly connected structure has emerged as a promising way to provide symptomatic relief not yet obtained by usual treatments. Here we review the most relevant papers published to date, the limitations and gaps in literature, discuss why several papers have failed in showing efficacy, and present a new way of stimulating the cerebellum. References for this critique review were identified by searches on PubMed for the terms “Parkinson’s disease”, “ataxia”, “dystonia”, “tremor”, and “dyskinesias” in combination with the type of stimulation and the stimulation site. Studies conducted thus far have shed light on the potential of cerebellar neuromodulation for attenuating symptoms in patients with some forms of isolated and combined dystonia, dyskinesia in Parkinson’s disease, and neurodegenerative ataxia. However, there is still a high heterogeneity of results and uncertainty about the possibility of maintaining long-term benefits. Because of the complicated architecture of the cerebellum, the modulation techniques employed may have to focus on targeting the activity of the cerebellar nuclei rather than the cerebellar cortex. Measures of cerebellar activity may reduce the variability in outcomes.

## 1. Introduction

Current neuromodulation techniques to treat Parkinson’s disease (PD), essential tremor, and isolated dystonia are mainly based on targeting deep basal ganglia nuclei. Despite well-defined benefits of such intervention, some symptoms, such as gait and balance impairments in PD, and complex syndromes, such as combined dystonia and cerebellar ataxia, are only marginally influenced by basal ganglia-based approaches, fueling the quest for novel targets to improve long-term control of these so far ill-controlled symptoms.

Traditionally, the study of the basal ganglia and thalamus have been used to map movement disorders into specific subcortical regions [1]. However, many neurologic symptoms correspond more closely to networks of connected distant regions [2]. Likewise, targeting other nodes of the movement circuitry could influence functionally and structurally interconnected regions, leading to new treatment targets for complex neurological syndromes [3].

In this scenario, the connectivity power of the cerebellum has motivated the study of its modulation among many teams worldwide, and it has been so far explored in a range of well-conducted preclinical and clinical studies [4,5]. The appeal of the cerebellum for neuromodulation strategies is easy to understand: it is a fascinating structure that boasts more neurons than all of the other brain regions combined, and it is implicated in virtually all movement disorders known to date.

## 2. Search Strategy and Selection Criteria

References for this article were identified by searches on PubMed, and references from relevant articles. We searched for the terms “Parkinson’s disease”, “ataxia”, “dystonia”, “tremor”, and “dyskinesias” in combination with terms describing the type of stimulation (transcranial magnetic stimulation (TMS), transcranial direct current stimulation (tDCS), or deep brain stimulation (DBS)) and the stimulation site (cerebellum, posterior cranium fossa, or cerebellar nuclei). Information was extracted from each included trial on the (1) characteristics of study population (number, type of movement disorder, and severity of disease), (2) type of intervention, (3) intervention targets, (4) assessment time points, (5) side effects, and (6) outcomes. There were no language restrictions. The final reference list was generated on the basis of relevance to the topics covered in this article.

## 3. A Window to Connect the Whole Brain

**Figure 1:**
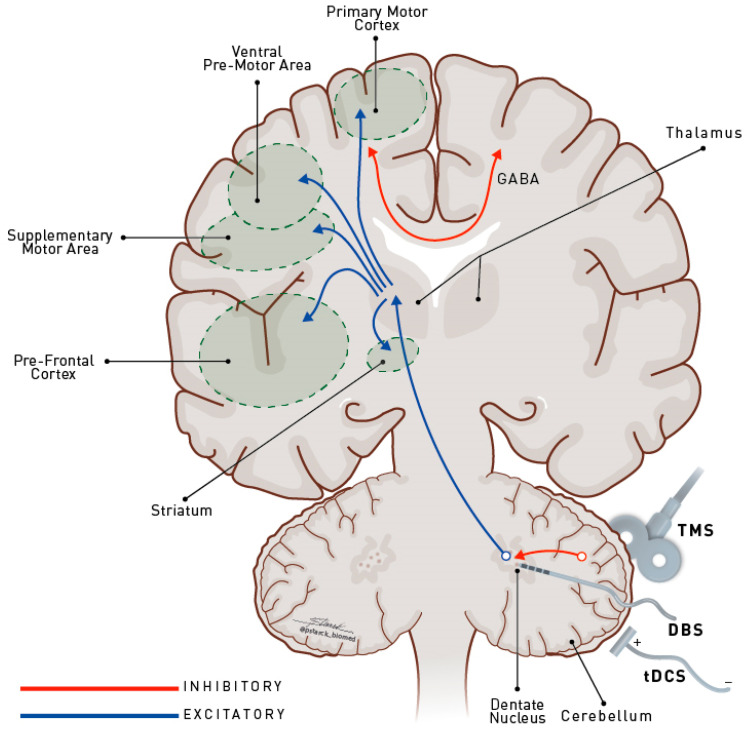
There is an intracortical inhibition between both M1 cortices that is related to maintaining the integrity of axial and limbs movements. The modulation of dentate nucleus activity through tDCS, TMS, or DBS could restore the changes in M1 cortical excitability that are present in some syndromes, such as degenerative ataxia, cerebellar stroke, and dystonia. Additionally, the recent disynaptic connection from the cerebellum to the striatum opens up the possibility of directly modulating aberrant electricity activity in the basal ganglia seen in a range of movement disorders. M1: primary motor cortex; tDCS: transcranial direct current stimulation; TMS: transcranial magnetic stimulation; DBS: deep brain stimulation (adapted from França et al. [9]).

There is growing evidence that the ideal area for neuromodulation is rather heterogenous within the same “anatomical” target, and influencing the activity of subregions within the same target may provide different clinical results based on the distinct, functionally related networks [2]. For example, parkinsonian patients respond better to subthalamic deep brain stimulation (STN DBS) when the stimulation site is functionally connected to the supplementary motor area [2], while tics in patients with Gilles de la Tourette syndrome are better controlled when the frontal middle gyrus and cingulate are more intensely connected with thalamic stimulation [6]. Cerebellar modulation opens the possibility of modulating the dentato-thalamic pathway and the activities of distant areas, such as the prefrontal, parietal and temporal lobes, and basal ganglia, due to its largely cortical and subcortical connections [5] (Figure 1).

In primates, deep cerebellar nuclei exert a primarily facilitatory effect on excitability in the contralateral primary motor cortex (M1) through dentothalamocortical projections [7]. In healthy individuals, a transcranial magnetic stimulation (TMS) pulse delivered to the cerebellum a few milliseconds before a TMS pulse is administered to the contralateral M1 results in M1 inhibition, revealed by decreased motor-evoked potential amplitude responses (cerebellar brain inhibition) [8]. This is thought to occur due to disruption of the tonic cerebellar facilitatory output to the contralateral M1 under physiologic conditions [3,8]. This normal balance is perturbed by disease (i.e., degenerative ataxia, cerebellar stroke, and dystonia) [3,5,8], and may affect the physiologic interhemispheric inhibition (how both M1s interact with one another) (Figure 1). For example, abnormal asymmetry in cortical excitability between the right and left hemispheres has been related to the motor impairment seen in cerebellar ataxia [7,8], which was normalized after cerebellar stimulation, improving the symptoms. This network connectivity allows for the construction of models to explain how the modulation of a normal or diseased cerebellum can restore the function of a dysfunctional network due to neurodegeneration or lesions to one of its hubs [3].

## 4. Why Target the Cerebellum in Movement Disorders?

Neuroanatomical studies using transneuronal virus tracers in monkeys have demonstrated that substantial interactions exist between the basal ganglia and the cerebellum [10]. Probabilistic diffusion tractography has confirmed that dentato–thalamo–striato–pallidal and subthalamo–cerebellar connections also exist in the human brain [11]. Consequently, abnormal cerebellar output could alter activity in the basal ganglia and drive aberrant electricity activity, causing or worsening movement disorders [12]. Furthermore, basal ganglia activity may influence the cerebellum via projections of the subthalamic nucleus to pontine nuclei, which then project to the cerebellum, demonstrating bidirectional connections between these structures [12]. Functional perturbation in these connections may underlie the pathophysiology of dystonia, PD, and spinocerebellar ataxia [3].

It has been shown, for example, that abnormal bursts of cerebellar electroencephalographic activity are correlated with dystonic postures [13]. Notably, disruption of the disynaptic connections between the cerebellum and basal ganglia have been shown to alleviate dystonia in a mouse model [13]. Furthermore, studies of patients with genetic isolated dystonia DYT-TOR1A (formerly known as DYT1) have shown that patients exhibit specific changes in cerebellar connectivity compared with controls and unaffected mutation carriers [14]. Because the non-responder rate of globus pallidus internus DBS in isolated dystonia can reach 25% in clinical trials [15], and patients with combined dystonia, such as cerebral palsy, are typically poor responders to pallidal stimulation [15], novel primary targets for dystonia or rescue treatments must be explored.

In PD, cerebellar brain inhibition is reduced, suggesting that cerebellar function or transmission along the cerebellothalamocortical pathway is compromised [16]. Additionally, PD patients have deficient short-latency and long-lasting cerebellar–thalamocortical inhibitory interactions [3]. Previous TMS studies for tremor have suggested that the cerebello–thalamo–cortical circuit may play a pivotal role in the pathogenesis of parkinsonian tremor, and neuroimaging studies have found hyperactivity in the cerebellum in PD [3,5].

Besides its widespread connections, unlike the deeply located basal ganglia and brainstem targets already tested for DBS, the cerebellum can be preoperatively and non-invasively modulated. Thus far, except for the preoperative use of levodopa challenge prior to surgery in PD, there are no other consistent ways of preoperatively predicting surgery outcomes.

## 5. What Recent Positive Studies Have Revealed

Cerebellar stimulation could alleviate some aspects of dystonia, especially those related to posture, as has been recently shown in rodents [17]. There is also evidence from clinical studies that TMS of the cerebellum may alleviate symptoms in cervical dystonic patients (Table 1) [12]. Cerebellar anodal transcranial direct current stimulation (tDCS) improved handwriting and circle-drawing tasks in patients with writing dystonia [18]. Another study demonstrated that bilateral deep anterior cerebellar stimulation in patients with secondary dystonia reduces both dystonic symptoms and spasticity [19]. More recently, a patient with generalized fixed dystonia, having failed bilateral pallidotomy, presented significant benefits after high-frequency bilateral superior cerebellar peduncles and dentate nuclei DBS, highlighting that cerebellar DBS may be a new option for fixed dystonia, refractory to classical DBS approaches [20]. In PD, cerebellar continuous theta burst stimulation has been found to change local intracortical circuits in the primary motor cortex and reduce levodopa-induced dyskinesias [21].

To date, most trials involving ataxic patients have focused on degenerative ataxias. Studies have identified temporary and long-lasting (3 months) functional improvement after cerebellar tDCS in patients with ataxia [3,5,37]. Recently, we have demonstrated in a clinical trial that cerebellar TMS using a deep coil improved ataxia in patients with spinocerebellar ataxia type 3 (SCA3), multiple-system atrophy, and post-lesion ataxia (post-stroke or neurosurgery) [9].

Regarding invasive stimulation, low-frequency DBS of the dentate nucleus has been applied in a rat model of neurogenerative ataxia [4]. A frequency of 30 Hz improved motor symptoms, such as ataxia and tremor, and high-frequency stimulation worsened incoordination. This study is probably the most significant in suggesting that the “hot spot” for stimulation would be located at the dentate nucleus. The authors found that the dorsal part of the nucleus was the most effective target for stimulation. In humans, two case reports demonstrated improvement in ataxia after cerebellar DBS in SCA3 and post-lesion ataxia [45,46,47].

Overall, studies conducted thus far, despite having methodological flaws, have shed light on the possibility of relieving symptoms in patients with some forms of dystonia, dyskinesia in PD, and neurodegenerative ataxia.

## 6. Playing Devil’s Advocate

The recent inclusion of cerebellar stimulation as an option to treat refractory cerebellar ataxia is likely due to the absence of any safer, better treatment option, along with non-invasive stimulation being safe in these settings. However, despite some good outcomes of cerebellar modulation in treating movement disorders in general, there is still a high heterogeneity of parameters employed in the available studies. The best stimulation paradigms and the best profiles of responders are still coupled with uncertainties about the possibility of maintaining long-term benefits [5], which makes it still difficult to currently advise the cerebellum as a new target. Although neurodegenerative ataxia remains orphaned of disease-modifying therapies, current results from cerebellar neuromodulation approaches may suffer from publication bias of positive results and small sample sizes, besides suboptimal blinding. Also, most studies have focused on stimulating still-imprecise areas within the cerebellar cortices, using tDCS or figure-of-eight TMS (i.e., superficial stimulations), with the goal of having an indirect effect on cerebellar–cortex connections [5]. There is currently a lack of information about the specific effects of cerebellar–cortex stimulation on various groups of neurons (e.g., Purkinje neurons, inhibitory interneurons of the cerebellar cortex, and granule cells) and afferent pathways (e.g., mossy fibers and climbing fibers) [5]. Because the cerebellum has a highly convoluted and completely different cytoarchitecture than the neocortex, generalizations of current density and geometry obtained from neocortical stimulation by TMS and tDCS are at least over-optimistic. This lack of specificity makes us rethink whether we are applying the stimulus at the right spot. Because of the complicated architecture of the cerebellum, the focus perhaps should shift from modulating the cerebellar cortex to targeting its output nuclei. This strategy could increase the stimulation’s efficiency and reduce variability in the outcomes of cortical stimulation. On an organizational level, the fibers from the cerebellar nuclei directly regulate movement commands in the spinal cord and brainstem, increase motor signals in the cerebral cortex, and modulate signals for adaptive learning via connections to the inferior olive. Direct stimulus to the dentate nucleus via a double-cone coil TMS (which allows for the stimulation of deep structures) [8] and DBS could be more precise, resetting abnormal firing oscillations or enhancing cerebellar output activity, depending on the parameters [4].

Several studies using cerebellar tDCS have compared both anodal and cathodal stimulation with a sham condition. Varying results have been obtained. Most of the studies report a different effect for anodal and cathodal tDCS. Some studies [48] have reported increased cerebellar brain inhibition following anodal stimulation applied over the cerebellar cortex. On the other hand, cathodal stimulation has reduced cerebellar brain inhibition. Two studies found the opposite effect [49,50]. Other studies did not find any effect after either stimulation type [24,51]. Additionally, many studies evaluating the effects of cerebellar cortical stimulation have been negative for motor outcomes in PD [23], essential tremor [42], and dystonia [27] or these studies found considerable side effects [22]. A recent, randomized, sham-controlled study failed to show the efficacy of figure-of-eight TMS over the cerebellum in 22 essential tremor patients [44]. Again, the absence of Magnetic Resonance Imaging-navigated systems and the superficial TMS stimulation applied bring doubt upon which regions of this overpopulated brain area we are stimulating.

## 7. So, What Is Next?

It is still unknown exactly what type of activity we are triggering when we stimulate the dentate nucleus. There are probable antidromic effects within the cerebellar cortex, but it would be interesting to test whether there are different responses within the thalamus and other downstream targets, depending on the topography stimulated. If this is true, one must consider the possibility that direct dentate nucleus stimulation could have variable effects, according to which specific regions are recruited [5]. Evidence suggests that the hot spot of modulation is likely located in more dorsal parts of the dentate nucleus, the presumed motor domain [4]. The study of the volume of tissue activated through DBS contacts can represent a powerful research platform to study connectomics from distributed brain networks in the “human connectome” [2].

Additionally, knowledge about modifications in the cerebellum circuitry in each disease, both neuropathological and functional, should help practitioners make decisions about the ideal type of stimuli to apply over the cerebellum. Such work is necessary before proceeding to multicenter clinical trials. Measures of cerebellar activity using functional and Positron Emission Tomography studies and cortical excitability may help with this issue.

Whether the “little brain” will be a primary or a rescue/adjunctive therapy in movement disorders remains an open question. It could perhaps be an alternative target for patients for whom the risk of surgery is high. Substantial changes in clinical practice are often tied to apprehension, but remarkable benefits may arise from innovations.

## Figures and Tables

**Table 1:** Clinical trials of cerebellar neuromodulation for Parkinson’s disease, dystonia, cerebellar ataxia, and essential tremor.

Author, Year	Study Design	Diagnosis, *n*	Intervention	Main Clinical Findings	Class of Evidence
**Parkinson’s disease**					
Koch et al., 2009 [21]	Double-blind, sham-controlled, crossover	PD with dyskinesias, 10	rTMS (cTBS) single session with figure-of-eight coil	Decrease in waking time spent as ON with dyskinesias	III
Minks et al., 2011 [22]	Single-blind, sham-controlled, crossover	PD, 20	One Hz rTMS, single session, with a double-cone coil	Improvement in gross upper limb movement; worsening in fine motor finger and hand function	III
Bologna et al., 2015 [23]	Double-blind, sham-controlled, crossover	PD, 13 + healthy controls, 10	Unilateral TMS (cTBS) single session with figure-of-eight coil	No changes in tremor amplitude, frequency, or magnitude	III
Ferrucci et al., 2016 [24]	Double-blind, sham-controlled, crossover	PD with dyskinesias, 9	Two mA anodal tDCS, five sessions	Improvement in UPDRS IV (dyskinesias section)	III
Sanna et al., 2020 [25]	Double-blind, sham-controlled, crossover	PD with dyskinesias, 11	rTMS (cTBS) single session with circular coil	Decrease in dyskinesias and serum BDNF in active group	II
Workman et al., 2020 [26]	Double-blind, sham-controlled, crossover	PD, 7	Two or 4 mA, unilateral or bilateral tDCS single session	Significant improvement in balance score in bilateral 4 mA group against sham; no gait improvement	II
**Dystonia**					
Sadnicka et al., 2014 [27]	Single-blinded, sham controlled with crossover	WC, 10	Two mA ipsilateral anodal tDCS, single session	No subjective improvement or changes in the WCRS or timed writing assessment	III
Koch et al., 2014 [28]	Double-blind, sham-controlled	CD, 18 (9 active; 9 sham)	Bilateral rTMS (cTBS), 10 sessions	Small but significant clinical improvement as measured by the TWSTRS of approximately 15%	III
Bradnam et al., 2015 [18]	Double-blind, sham-controlled, crossover	FHD, 8 (WC = 5; MD = 3); healthy controls, 8	Two mA anodal/cathodal tDCS, single session	No change in clinical outcomes	II
**Cerebellar ataxia**					
Shiga et al., 2002 [29]	Double-blind, sham-controlled	Spinocerebellar degeneration, 74 (39 active, 35 sham)	Single-pulse TMS, 21 sessions with circular coil	Improvement in 10 m time, 10 m steps, tandem steps. and standing capacities, especially in the cerebellar type	III
Ihara et al., 2005 [30]	Single-blind, uncontrolled	Spinocerebellar degeneration, 20	Single-pulse TMS, 24 sessions with figure-of-eight coil	Improvement in ataxia (ICARS)	III
Grimaldi and Manto et al., 2013 [31]	Single-blind, sham-controlled, crossover	Varied cerebellar ataxias, 9	One mA right anodal tDCS, single session	No change in posturography or upper limb dexterity	III
Bonnì et al., 2014 [32]	Open label	Posterior circulation stroke with ataxia, 6	rTMS (iTBS, ipsilateral), 10 sessions with figure-of-eight coil + physical therapy	Ataxia improvement (MICARS), especially posture and gait subscales	IV
Kim et al., 2014 [33]	Double-blind, sham-controlled	Posterior circulation stroke with ataxia, 32	One Hz ipsilateral rTMS, five sessions with figure-of-eight coil	Improvement in the 1 0m walk test 1 month after; balance improved after 5 days and after 1 month	III
Benussi et al., 2015 [34]	Double-blind, sham-controlled, crossover	Varied cerebellar ataxias, 19	Two mA anodal tDCS, single session	Improvement in ataxia (SARA and ICARS), hand dexterity, and gait	III
Grecco et al., 2017 [35]	Single-blind, sham-controlled, crossover	Ataxic cerebral palsy, 6	One mA anodal tDCS, 10 sessions + treadmill training	Improvement in hip oscillation during eyes-closed gait (stabilometric evaluation)	III
Benussi et al., 2017 [36]	Double-blind, sham-controlled	Varied neurodegenerative ataxias, 20; healthy controls, 10	Two mA anodal tDCS, 10 sessions	Improvement lasting at least 3 months in SARA, ICARS, gait, and hand dexterity (in non-dominant hand)	III
Benussi et al., 2018 [37]	Double-blind, sham-controlled crossover	Varied neurodegenerative ataxias, 20	Two mA anodal tDCS (cerebellum) and 2 mA cathodal tDCS (spinal cord), 10 sessions	Improvement lasting at least 3 months in SARA, ICARS, gait, hand dexterity, and quality of life	II
Manor et al., 2019 [38]	Double-blind, sham-controlled	Spinocerebellar ataxia, 20	Single-pulse TMS, 20 sessions with circular coil	Improvement only in stance sub-score of SARA and standing postural sway metrics	II
França et al., 2020 [9]	Double-blind, sham-controlled, crossover	Spinocerebellar ataxia type 3, 9; multiple system atrophy cerebellar type, 8; post-lesion ataxia, 7	One Hz unilateral rTMS, 10 sessions with double-cone coil	Improvement in SARA and ICARS	II
**Essential tremor**					
Gironell et al., 2002 [39]	Double-blind, sham-controlled, crossover (washout 1 week)	ET, 10	One Hz rTMS, single session with butterfly coil	Tremor improvement according to the FTM (17%), and accelerometry evaluation on the 5 min assessment	II
Avanzino et al., 2009 [40]	Open label in five patients, and single-blind, sham-controlled, crossover in seven patients	ET, 10 + healthy controls, 11	One Hz right rTMS, single session with figure-of-eight coil	Decrease of TD values; increase of ITI values and decrease of the coefficient of variation of ITI; no change in frequency or magnitude of accelerometer signal, and no change in tremor (FTM)	IV
Popa et al., 2013 [41]	Open label	ET, 11; healthy controls, 11	One Hz rTMS, five sessions with figure-of-eight coil	Tremor improvement that built up until day 12 and persisted for 3 weeks (FTM); decrease in tremor amplitude.	IV
Gironell et al., 2014 [42]	Double-blind, sham-controlled crossover	ET, 10	Two mA cathodal tDCS, 10 sessions	No acute or long-lasting benefit (FTM and accelerometric recordings)	III
Bologna et al., 2015 [43]	Double-blind, sham-controlled, crossover	ET, 16; healthy controls, 11	rTMS (cTBS), single session with eight-shaped coil	No change in tremor severity and reaching movements (FTM and accelerometer)	III
Shin et al., 2019 [44]	Single-blind, sham-controlled	ET, 22 (12 active, 10 sham)	One Hz rTMS, five sessions with figure-of-eight coil	Improvement in tremor immediately after (33% active × 20% sham, according to FTM) and 4 weeks after (31% active × 17% sham); no significant difference between groups; no improvement in functions of daily lives	III

Abbreviations: BDNF: brain-derived neurotrophic factor; CD: cervical dystonia; cTBS: continuous theta burst stimulation; ET: essential tremor; FHD: focal hand dystonia; FTM: Fahn Tolosa Marin Tremor Rating Scale; ICARS: International Cooperative Ataxia Rating; iTBS: intermittent theta burst stimulation; ITI: inter-tapping interval; MD: musician’s dystonia; MICARS: Modified International Cooperative Ataxia Rating Scale; PD: Parkinson’s disease; rTMS: repetitive transcranial magnetic stimulation; SARA: scale for the assessment and rating of ataxia; TD: touch duration; tDCS: transcranial direct current stimulation; TMS: transcranial magnetic stimulation; TWSTRS: Toronto Western Spasmodic Torticollis Rating Scale; UPDRS: Unified Parkinson’s Disease Rating Scale; WC: writer’s cramp; WCRS: writer’s cramp rating scale.

## References

[1] Bostan A.C., Strick P.L. (2018). The basal ganglia and the cerebellum: Nodes in an integrated network. Nat. Rev. Neurosci..

[2] Horn A., Reich M., Vorwerk J., Li N., Wenzel G., Fang Q., Schmitz-Hübsch T., Nickl R., Kupsch A., Volkmann J. (2017). Connectivity Predicts deep brain stimulation outcome in Parkinson disease. Ann. Neurol..

[3] França C., de Andrade D.C., Teixeira M.J., Galhardoni R., Silva V., Barbosa E.R., Cury R.G. (2018). Effects of cerebellar neuromodulation in movement disorders: A systematic review. Brain Stimul..

[4] Anderson C.J., Figueroa K.P., Dorval A.D., Pulst S.M. (2019). Deep cerebellar stimulation reduces ataxic motor symptoms in the shaker rat. Ann. Neurol..

[5] Miterko L.N., Baker K.B., Beckinghausen J., Bradnam L.V., Cheng M.Y., Cooperrider J., DeLong M.R., Gornati S.V., Hallett M., Heck D.H. (2019). Consensus Paper: Experimental Neurostimulation of the Cerebellum. Cerebellum.

[6] Brito M., Teixeira M.J., Mendes M.M., França C., Iglesio R., Barbosa E.R., Cury R.G. (2019). Exploring the clinical outcomes after deep brain stimulation in Tourette syndrome. J. Neurol. Sci..

[7] Da Guarda S.N.F., Cohen L.G., da Cunha Pinho M., Yamamoto F.I., Marchiori P.E., Scaff M., Conforto A.B. (2010). Interhemispheric asymmetry of corticomotor excitability after chronic cerebellar infarcts. Cerebellum.

[8] Cury R.G., Teixeira M.J., Galhardoni R., Barboza V.R., Alho E., Seixas C.M., Lepski G., de Andrade D.C. (2015). Neuronavigation-guided transcranial magnetic stimulation of the dentate nucleus improves cerebellar ataxia: A sham-controlled, double-blind n = 1 study. Parkinsonism Relat. Disord..

[9] França C., de Andrade D.C., Silva V., Galhardoni R., Barbosa E.R., Teixeira M.J., Cury R.G. (2020). Effects of cerebellar transcranial magnetic stimulation on ataxias: A randomized trial. Parkinsonism Relat. Disord..

[10] Hoshi E., Tremblay L., Féger J., Carras P.L., Strick P.L. (2005). The cerebellum communicates with the basal ganglia. Nat. Neurosci..

[11] Pelzer E.A., Hintzen A., Goldau M., von Cramon D.Y., Timmermann L., Tittgemeyer M. (2013). Cerebellar networks with basal ganglia: Feasibility for tracking cerebello-pallidal and subthalamo-cerebellar projections in the human brain. Eur. J. Neurosci..

[12] Tewari A., Fremont R., Khodakhah K. (2017). It’s not just the basal ganglia: Cerebellum as a target for dystonia therapeutics. Mov. Disord. Off. J. Mov. Disord. Soc..

[13] Calderon D.P., Fremont R., Kraenzlin F., Khodakhah K. (2011). The neural substrates of rapid-onset Dystonia-Parkinsonism. Nat. Neurosci..

[14] Argyelan M., Carbon M., Niethammer M., Ulug A.M., Voss H.U., Bressman S.B., Dhawan V., Eidelberg D. (2009). Cerebellothalamocortical connectivity regulates penetrance in dystonia. J. Neurosci. Off. J. Soc. Neurosci..

[15] Cury R.G., Kalia S.K., Shah B.B., Jimenez-Shahed J., Prashanth L.K., Moro E. (2018). Surgical treatment of dystonia. Expert Rev. Neurother..

[16] Ni Z., Pinto A.D., Lang A.E., Chen R. (2010). Involvement of the cerebellothalamocortical pathway in Parkinson disease. Ann. Neurol..

[17] White J.J., Sillitoe R.V. (2017). Genetic silencing of olivocerebellar synapses causes dystonia-like behaviour in mice. Nat. Commun..

[18] Bradnam L.V., Graetz L.J., McDonnell M.N., Ridding M.C. (2015). Anodal transcranial direct current stimulation to the cerebellum improves handwriting and cyclic drawing kinematics in focal hand dystonia. Front. Hum. Neurosci..

[19] Sokal P., Rudaś M., Harat M., Szylberg Ł., Zieliński P. (2015). Deep anterior cerebellar stimulation reduces symptoms of secondary dystonia in patients with cerebral palsy treated due to spasticity. Clin. Neurol. Neurosurg..

[20] Horisawa S., Arai T., Suzuki N., Kawamata T., Taira T. (2019). The striking effects of deep cerebellar stimulation on generalized fixed dystonia: Case report. J. Neurosurg..

[21] Koch G., Brusa L., Carrillo F., Lo Gerfo E., Torriero S., Oliveri M., Mir P., Caltagirone C., Stanzione P. (2009). Cerebellar magnetic stimulation decreases levodopa-induced dyskinesias in Parkinson disease. Neurology.

[22] Minks E., Mareček R., Pavlík T., Ovesná P., Bareš M. (2011). Is the cerebellum a potential target for stimulation in Parkinson’s disease? Results of 1-Hz rTMS on upper limb motor tasks. Cerebellum.

[23] Bologna M., Di Biasio F., Conte A., Iezzi E., Modugno N., Berardelli A. (2015). Effects of cerebellar continuous theta burst stimulation on resting tremor in Parkinson’s disease. Parkinsonism Relat. Disord..

[24] Ferrucci R., Cortese F., Bianchi M., Pittera D., Turrone R., Bocci T., Borroni B., Vergari M., Cogiamanian F., Ardolino G. (2016). Cerebellar and Motor Cortical Transcranial Stimulation Decrease Levodopa-Induced Dyskinesias in Parkinson’s Disease. Cerebellum.

[25] Sanna A., Follesa P., Puligheddu M., Cannas A., Serra M., Pisu M.G., Dagostino S., Solla P., Tacconi P., Marrosu F. (2020). Cerebellar continuous theta burst stimulation reduces levodopa-induced dyskinesias and decreases serum BDNF levels. Neurosci. Lett..

[26] Workman C.D., Fietsam A.C., Uc E.Y., Rudroff T. (2020). Cerebellar Transcranial Direct Current Stimulation in People with Parkinson’s Disease: A Pilot Study. Brain Sci..

[27] Sadnicka A., Hamada M., Bhatia K.P., Rothwell J.C., Edwards M.J. (2014). Cerebellar stimulation fails to modulate motor cortex plasticity in writing dystonia. Mov. Disord. Off. J. Mov. Disord. Soc..

[28] Koch G., Porcacchia P., Ponzo V., Carrillo F., Cáceres-Redondo M.T., Brusa L., Desiato M.T., Arciprete F., Di Lorenzo F., Pisani A. (2014). Effects of two weeks of cerebellar theta burst stimulation in cervical dystonia patients. Brain Stimul..

[29] Shiga Y. (2002). Transcranial magnetic stimulation alleviates truncal ataxia in spinocerebellar degeneration. J. Neurol. Neurosurg. Psychiatry.

[30] Ihara Y., Takata H., Tanabe Y., Nobukuni K., Hayabara T. (2005). Influence of repetitive transcranial magnetic stimulation on disease severity and oxidative stress markers in the cerebrospinal fluid of patients with spinocerebellar degeneration. Neurol. Res..

[31] Grimaldi G., Manto M. (2013). Anodal transcranial direct current stimulation (tDCS) decreases the amplitudes of long-latency stretch reflexes in cerebellar ataxia. Ann. Biomed. Eng..

[32] Bonnì S., Ponzo V., Caltagirone C., Koch G. (2014). Cerebellar theta burst stimulation in stroke patients with ataxia. Funct. Neurol..

[33] Kim W.-S., Jung S.H., Oh M.K., Min Y.S., Lim J.Y., Paik N.-J. (2014). Effect of repetitive transcranial magnetic stimulation over the cerebellum on patients with ataxia after posterior circulation stroke: A pilot study. J. Rehabil. Med..

[34] Benussi A., Koch G., Cotelli M., Padovani A., Borroni B. (2015). Cerebellar transcranial direct current stimulation in patients with ataxia: A double-blind, randomized, sham-controlled study. Mov. Disord. Off. J. Mov. Disord. Soc..

[35] Grecco L.A.C., Oliveira C.S., de Almeida Carvalho Duarte N., Lima V.L.C.C., Zanon N., Fregni F. (2017). Cerebellar transcranial direct current stimulation in children with ataxic cerebral palsy: A sham-controlled, crossover, pilot study. Dev. Neurorehabilit..

[36] Benussi A., Dell’Era V., Cotelli M.S., Turla M., Casali C., Padovani A., Borroni B. (2017). Long term clinical and neurophysiological effects of cerebellar transcranial direct current stimulation in patients with neurodegenerative ataxia. Brain Stimul..

[37] Benussi A., Dell’Era V., Cantoni V., Bonetta E., Grasso R., Manenti R., Cotelli M., Padovani A., Borroni B. (2018). Cerebello-spinal tDCS in ataxia: A randomized, double-blind, sham-controlled, crossover trial. Neurology.

[38] Manor B., Greenstein P.E., Davila-Perez P., Wakefield S., Zhou J., Pascual-Leone A. (2019). Repetitive Transcranial Magnetic Stimulation in Spinocerebellar Ataxia: A Pilot Randomized Controlled Trial. Front. Neurol..

[39] Gironell A., Kulisevsky J., Lorenzo J., Barbanoj M., Pascual-Sedano B., Otermin P. (2002). Transcranial magnetic stimulation of the cerebellum in essential tremor: A controlled study. Arch. Neurol..

[40] Avanzino L., Bove M., Tacchino A., Ruggeri P., Giannini A., Trompetto C., Abbruzzese G. (2009). Cerebellar involvement in timing accuracy of rhythmic finger movements in essential tremor. Eur. J. Neurosci..

[41] Popa T., Russo M., Vidailhet M., Roze E., Lehéricy S., Bonnet C., Apartis E., Legrand A.P., Marais L., Meunier S. (2013). Cerebellar rTMS stimulation may induce prolonged clinical benefits in essential tremor, and subjacent changes in functional connectivity: An open label trial. Brain Stimul..

[42] Gironell A., Martínez-Horta S., Aguilar S., Torres V., Pagonabarraga J., Pascual-Sedano B., Ribosa-Nogué R. (2014). Transcranial direct current stimulation of the cerebellum in essential tremor: A controlled study. Brain Stimul..

[43] Bologna M., Rocchi L., Leodori G., Paparella G., Conte A., Kahn N., Fabbrini G., Berardelli A. (2015). Cerebellar continuous theta burst stimulation in essential tremor. Cerebellum.

[44] Shin H.-W., Hallett M., Sohn Y.H. (2019). Cerebellar repetitive transcranial magnetic stimulation for patients with essential tremor. Parkinsonism Relat. Disord..

[45] Teixeira M.J., Cury R.G., Galhardoni R., Barboza V.R., Brunoni A.R., Alho E., Lepski G., de Andrade D.C. (2015). Deep brain stimulation of the dentate nucleus improves cerebellar ataxia after cerebellar stroke. Neurology.

[46] Cury R.G., França C., Barbosa E.R., Galhardoni R., Lepski G., Teixeira M.J., Ciampi de Andrade D. (2019). Dentate nucleus stimulation in a patient with cerebellar ataxia and tremor after cerebellar stroke: A long-term follow-up. Parkinsonism Relat. Disord..

[47] Cury R.G., França C., Silva V., Barbosa E.R., Capato T.T.C., Lepski G., Duarte K.P., Teixeira M.J., Ciampi de Andrade D. (2019). Effects of dentate nucleus stimulation in spinocerebellar ataxia type 3. Parkinsonism Relat. Disord..

[48] Galea J.M., Jayaram G., Ajagbe L., Celnik P. (2009). Modulation of cerebellar excitability by polarity-specific noninvasive direct current stimulation. J. Neurosci. Off. J. Soc. Neurosci..

[49] Bocci T., Santarcangelo E., Vannini B., Torzini A., Carli G., Ferrucci R., Priori A., Valeriani M., Sartucci F. (2015). Cerebellar direct current stimulation modulates pain perception in humans. Restor. Neurol. Neurosci..

[50] Panouillères M.T.N., Miall R.C., Jenkinson N. (2015). The role of the posterior cerebellum in saccadic adaptation: A transcranial direct current stimulation study. J. Neurosci. Off. J. Soc. Neurosci..

[51] Sadnicka A., Kassavetis P., Saifee T.A., Pareés I., Rothwell J.C., Edwards M.J. (2013). Cerebellar transcranial direct current stimulation does not alter motor surround inhibition. Int. J. Neurosci..

